# Aeromonas sobria peritonitis in a peritoneal dialysis (PD) patient: a case report and review of the literature

**DOI:** 10.1186/s12882-019-1361-7

**Published:** 2019-05-20

**Authors:** Panai Song, Jun Deng, Tao Hou, Xiao Fu, Lei Zhang, Lin Sun, Yinghong Liu

**Affiliations:** 1 Department of Nephrology, Second Xiangya Hospital, Central South University, No.139, Renmin Middle Road, Changsha, 410011 Hunan China; 2 Department of Nephrology, Fourth Hospital of Changsha, Changsha, 410006 Hunan China; 3 Department of Oncology, Second Xiangya Hospital, Central South University, Changsha, 410011 Hunan China

**Keywords:** Peritoneal dialysis (PD)-related peritonitis, Aeromonas sobria, Stinky tofu, Antibiotics

## Abstract

**Background:**

Peritonitis is a common cause of catheter removal and mortality in the patient undergoing peritoneal dialysis (PD). Various pathogenic organisms have been identified as the etiology of PD-related peritonitis, among which Aeromonas sobria is a rare one. Several studies have indicated that Aeromonas sobria might be of particular clinical significance because of its enterotoxin production. We here present a case of peritonitis due to Aeromonas sobria in a PD patient and review of the related literature.

**Case presentation:**

A 37-year-old man with chronic renal failure who was secondary to chronic glomerulonephritis had been on PD for approximately 6 months without any episode of peritonitis. In July 2015, he was admitted to the hospital for fever, vomiting, abdominal pain, diarrhea and cloudy dialysate several hours after eating stinky tofu. The peritoneal effluent culture yielded Aeromonas sobria. The patient was given intraperitoneal amikacin and intravenous levofloxacin for 10 days. And the patient’s symptoms such as diarrhea, abdominal pain were relieved and the cloudy effluent turned to be clear. Unfortunately, peritoneal dialysis catheter was blocked because of fibrin clot formation in the setting of inflammation, and finally it was removed.

**Conclusions:**

Aeromonas species are rare causes of PD-related peritonitis, however they should not be ignored. Clinicians should be aware of monitoring the hygiene protocol and retraining patients at regular intervals, especially for such rare cases.

## Background

Peritonitis is not only a common complication in patients undergoing peritoneal dialysis (PD), but also a main cause of catheter removal and mortality in PD patients [1]. Various of pathogenic organisms, including Staphylococcus aureus, S.epidermidis and enterogenous bacteria, have been recognized as the pathogens of PD-related peritonitis, among which Aeromonas sobria is rare. Several studies have indicated that Aeromonas sobria may be of particular clinical significance because of its enterotoxin production. The Aeromonas species, are facultative anaerobic, rod-shaped, gram-negative microorganisms widely found in water, sewage and soil, and can also be isolated from varieties of foods including raw meats, sea foods and milk. Animals and humans are usually infected through the contaminated food [2, 3]. Aeromonas species may act as conditional pathogenic bacteria which can cause various infections including bacteremia, wound infections, skin and soft-tissue infections, pneumonia, endophthalmitis, endocarditis, meningitis, cholangitis, urinary tract infections, septic arthritis, osteomyelitis, and gastroenteritis, especially in immunocompromised patients [2, 4]. Aeromonas sobria, which belongs to the Aeromonas species, has seldom been reported in PD-related peritonitis. Here, we present a case of peritonitis caused by Aeromona sobria in a PD patient and review of the related literature.

## Case presentation

A 37-year old man with chronic renal failure who was secondary to chronic glomerulonephritis had been on PD for approximately 6 months without any episode of peritonitis. In July 2015, he was admitted to the hospital because of fever, vomiting, abdominal pain, diarrhea and cloudy dialysate several hours after eating stinky tofu. Physical examination showed: blood pressure was 175/97 mmHg, pulse was 90 beats per minute and body temperature was 39.1 °C, periumbilical tenderness, defense and rebound. No erythema and exudates were found around PD catheter exit site. Laboratory examinations revealed an increased white blood cell (WBC) count (14.22 × 10^9^ cells/L with 89.8% neutrophils). Hemoglobin was 110 g/L, albumin was 36.1 g/L, serum potassium was 2.86 mmol/L, and C-reactive protein was 67.5 mg/L. Dialysate leukocyte count was 12,800 × 10^6^ /L with 30% polymorphonuclear cells, indicating PD-related peritonitis. The first peritoneal effluent culture was obtained before initiation of antibiotics therapy (intraperitoneal teicoplanin 200 mg every other day and intravenous cefotiam 1000 mg twice daily for 8 days). After treatment, the patient’s fever and diarrhea were relieved. However, he still suffered from abdominal pain and the peritoneal effluent was still turbid. Analysis of dialysate for the second time showed that leukocyte count was 3200 × 10^6^ /L with 90% polymorphonuclear cells. Aeromona sobria was isolated from peritoneal effluent on the fifth day after the treatment, and drug sensitivity test showed that it is sensitive to amikacin, ceftazidime, cefepime, levofloxacin and meropenem, and resisted to ampicillin, cefotaxime, and piperacillin /tazobactam. Therefore, amikacin and levofloxacin (intraperitoneal amikacin 200 mg and intravenous levofloxacin 300 mg per day for 10 days) were prescript. The abdominal pain was relieved and peritoneal effluent turned to be clear gradually. Unfortunately, the peritoneal dialysis catheter was blocked because of fibrin clot formation in the setting of inflammation. Although urokinase was used to salvage the catheter, it was removed finally. The patient switched to hemodialysis and was discharged from hospital after recovery.

## Discussion and conclusions

Aeromonas is usually classified into four main species: *Aeromonas hydrophila*, Aeromonas caviae, *Aeromonas salmonicida* and Aeromonas sobria [5]. It’s commonly believed that the major virulence factors of Aeromonas species are haemolysins including enterotoxins, invasins, aerolysin, adhesins, proteases, phospholipase and lipase [4, 6]. Aeromonas sobria bacteremia is reported to have the highest mortality rate in this genus (Aeromonas sobria - 56%, Aeromonas hydrophyila - 33%, Aeromonas cavies - 17%) [7]. Aeromonas sobria may act as opportunistic pathogen that can cause bacteremia, intestinal and other extraintestinal infection, and can be isolated from sea water, soil, fish and many other food. Such infections occurred predominantly in patients with chronic hepatic disease, gastroenteritis, malignancy and immunocompromised status [4]. Although gastroenteritis is the most common infection of Aeromonas species, peritonitis caused by Aeromonas species are not uncommon, especially spontaneous bacterial peritonitis in patients with cirrhosis [8–10]. However, peritonitis episodes due to Aeromonas species have seldom been reported in PD patients [11–22] (Table 1). The most frequently isolated specie was *A. hydrophila* (10;71.4%), while *A. sobria* was reported owning higher virulence (1;7.1%). The pathogenic mechanism of Aeromonas peritonitis in patients undergoing PD could be associated with direct exposure to contaminated water. From the literature review (Table 1), we found that two of the cases may be possibility of water-related infection, however, such infection has rarely been proven. Another possible mechanism is transmural migration from the gastrointestinal tract to the blood. The outcomes of PD-related peritonitis caused by Aeromonas species are generally good; however, recurrent peritonitis can occur (21.4%). The reason of recurrence may be biofilm formation on the surfaces of catheter. Recently, Joana et al. [23] found that no major differences on microbial density of the catheter cultures were observed between the catheters removed due to infectious and non-infectious causes. However, microbial yields were higher on the cuffs of catheters removed due to infection, which indicated that microbial biofilm is universal in PD catheters with the subclinical menace. Cuffs colonization may significantly contribute to infection. In this sense, it would not be prudent to prophylactically remove the PD catheter in PD-related peritonitis patients.

**Table 1:** Cases of PD-related Peritonitis Caused by Aeromonas Species

Case/ Reference	Age	Sex	PD Duration (months)	Cause of ESRD	Reason of infection	Underlying condition	Aeromonas Species	Antibiotics	Outcome
1/ [11]	62	male	2	Nephrosclerosis	Exposure to goldfish water	Total gastrectomy due to perforation of hemorrhagic gastric ulcer 1 years ago	*A. hydrophila*	Cefazolin +cefepime	cure
2/ [12]	63	female	11	Systemic lupus erythematosus	Didn’t wear mask and omitted thorough hand washing during PD after she engaged in gardening	Systemic lupus erythematosus	A. hydrophila	Vancomycin + ceftazidime	cure
3/ [13]	44	female	5	Chronicglomerulonephritis	Peritoneal dialysis catheter dropped into the toilet	Poor hygiene	A. hydrophila	Cefazolin + ceftazidime	cure
4/ [14]	68	female	11	Diabetes	Ate raw or incompletely cooked fish	Chronic gastritis, duodenal ulcer	*A. salmonicida*	Cephradine +ceftazidime	cure
5/ [15]	54	female	19	Chronic glomerulonephritis	Gut bacterial translocation	Adenocarcinoma of the colon with distant metastasis	A. hydrophila	Cefazolin +tobramycin	Recurrence two times; shifted to hemodialysis
6/ [15]	70	male	22	Chronic renal failure	Gut bacterial translocation	Liver cirrhosis caused by hepatitis	A. hydrophila	Cefazolin +tobramycinj→ceftazidime	Recurrence 10 days later; then cure
7/ [16]	71	male	8	Congestive cardiomyopathy	fingernails were dirt after he engaged in gardening	nothing in particular	A. hydrophila	Vancomycin+ gentamicin+ ciprofloxacin	cure
8/ [17]	53	male	unknown	Chronic renal failure	unknown	unknown	A. caviae	Ampicillin+cefotaxime	cure
9/ [17]	55	male	unknown	Diabetes	unknown	unknown	A. hydrophila	Cefotaxime	cure
10/ [18]	52	female	10	unknown	Alcohol-base disinfectant spray also used for houseplant	nothing in particular	A. caviae	Vancomycin + aztreonam	cure
11/ [19]	53	female	18	Hypertension	Presence of the indwelling peritoneal catheter	Immunosuppressive therapy following cadaveric kidney transplantation	A. hydrophila	Cefotaxime	Recurrence 6 weeks later; then cure
12/ [20]	53	male	28	uknown	Transmural migration	Immunosuppressive therapy following cadaveric kidney transplantation	A. hydrophila	Ampicillin + flucloxacillin →cefotaxime	cure
13/ [21]	14	female	uknown	uknown	uknown	uknown	A. hydrophila	Ampicillin	Recovery; died fromuremia 1 week later
14/ [22]	70	male	36	Type 2 diabetes, hypertension	A laceration from flood disaster	Dyslipidemia and ischemic heart disease	*A. sobria*	Amikacin +levofloxacin	cure

Aeromonas peritonitis has an abrupt onset in most patients. In this case, the patient presented fever, vomiting, abdominal pain, diarrhea and cloudy peritoneal effluent several hours after eating stinky tofu. Stinky tofu, a kind of traditional Chinese food, is usually considered unhygienic. The tofu have to be placed in water for a long time to increase the unique smell. Human body may get infected after eating stinky tofu contaminated by Aeromonas sobria. Aeromonas can produce enterotoxin and hemolysin, causing gastrointestinal symptoms such as abdominal pain and diarrhea. Then the bacterial translocation plays a important role in the pathogenesis of PD-related peritonitis. Therefore, we speculated that the stinky tofu might be the source of infection.

Aeromonas sobria grow rapidly in summer [9]. The patient we reported here got infected in summer, and the anemia, low albumin, and immunosuppressive status of the patient increased the risk of opportunistic infection. Moreover, hypokalemia caused by vomiting and diarrhea not only decrease intestinal peristalsis, but also increase intestinal permeability, both of which facilitate bacterial translocation [24]. The increased cellulose exudation and fibrin clot formation in the setting of inflammation made peritoneal catheter blocked [25]. In consequence, the patient had to remove the catheter and switch to hemodialysis. Therefore, the patients undergoing PD should pay more attention not to eat any contaminated food, and avoid constipation in order to prevent from opportunistic bacterial infection.

Aeromonas species can produce β-lactamases which make most of them resistant to ampicillin, pencillin and first- or second-generation cephalosporins, while sensitive to third-generation cephalosporins, carbapenems, chloramphenicol, fluoroquinolones, and aminoglycosides [2, 4, 26–28], which is consistent with our drug sensitivity tests. However, our result indicated that Aeromonas sobria was resistant to cefotaxime which belongs to third-generation cephalosporins. Recent literature also suggests that Aeromonas species show an increasing trend of resistance to third-generation cephalosporins [2]. Amikacin and levofloxacin treatment were efficient in this case. The patient finally shifted to hemodialysis with the catheter removed. It’s noteworthy that the third-generation cephalosporins which is well known as the empirical approach to the therapy of bacterial infection caused by Aeromonas sobria may be ineffective sometimes.

In conclusion, Aeromonas species are rare causes of PD-related peritonitis. Which should not be ignored. Clinicians should be aware of monitoring the hygiene protocol and retraining patients, especially in such rare cases.

## Abbreviations

PD: Peritoneal dialysis
WBC: White blood cell
